# Mode of Delivery and Perinatal Outcomes in Pregnancies With Absent End-Diastolic Flow: A Prospective Observational Study

**DOI:** 10.7759/cureus.116723

**Published:** 2026-09-22

**Authors:** Udhaya Parivallal, Sujata Siwatch, Neelam Aggarwal, Rashmi Bagga, Subhas C Saha, Kanya Mukhopadhyay

**Affiliations:** 1 Urogynecology, KIMS Hospital, Secunderabad, IND; 2 Obstetrics and Gynecology, Post Graduate Institute of Medical Education and Research, Chandigarh, IND; 3 Neonatology (Pediatrics), Post Graduate Institute of Medical Education and Research, Chandigarh, IND

**Keywords:** absent end diastolic flow, cesarean birth, colored flow doppler ultrasound, perinatal morbidity, perinatal outcome

## Abstract

Background

Absent end-diastolic flow (AEDF) in the umbilical artery depicts an advanced stage of placental insufficiency. It is often associated with significant perinatal morbidity and mortality. While cesarean delivery is preferred, the evidence guiding the optimal mode of delivery remains limited.

Methods

This prospective observational study included 71 singleton pregnancies beyond 28 weeks with AEDF. Maternal, fetal, and delivery characteristics were recorded. The primary outcome was a composite perinatal outcome comprising cord pH <7.1, five-minute Apgar score <7, feeding intolerance, sepsis, necrotizing enterocolitis, or neonatal mortality.

Results

Overall, cesarean section was performed in 58 (81.7%) of cases. Of these, 33 underwent direct cesarean delivery without an attempted labor (categories 1 and 2), while 12 had elective surgery (categories 3 and 4). A trial of labor was attempted in 26 women, and 13 (50%) achieved vaginal deliveries. Adverse composite perinatal outcomes (APOs) occurred in 37 (52%) of the neonates, with 9 neonatal deaths. Lower birth weight and gestational age were significantly associated with adverse outcomes. Birth weight was an independent predictor in the multivariate analysis, whereas gestational age, duration of AEDF, and mode of delivery were not.

Conclusion

Perinatal outcomes in AEDF pregnancies are primarily influenced by birth weight. Mode of delivery did not independently affect adverse perinatal outcomes. A carefully selected trial of labor may be considered in appropriate clinical settings. Future adequately powered prospective comparative studies are needed to clarify the optimal mode of delivery in pregnancies complicated by AEDF.

## Introduction

Fetal Doppler surveillance plays a pivotal role in the management of fetuses with fetal growth restriction (FGR). The umbilical artery Doppler provides a hemodynamic assessment of placental dysfunction, and the presence of absent end-diastolic velocity (AEDV) reflects advanced placental vascular resistance. It is associated with increased perinatal risk [1,2]. Current guidelines generally recommend delivery at approximately 33-34 weeks for FGR complicated by persistent AEDV, balancing the risks of continued placental insufficiency against the morbidity associated with prematurity [1,3]. The FIGO recommendations acknowledge that the optimal timing and mode of delivery in severe early-onset FGR are based largely on expert consensus and observational evidence rather than randomized comparisons of delivery mode [2]. Although cesarean delivery is commonly preferred when severe early-onset FGR is complicated by AEDV or other evidence of fetal compromise because of concerns regarding fetal intolerance of labor, FGR itself is not an absolute indication for cesarean delivery [2].

The GRIT and TRUFFLE trials primarily addressed the balance between continuing pregnancy and delivering a growth-restricted fetus, rather than comparing planned vaginal and cesarean delivery [4,5]. Consequently, direct evidence regarding whether planned cesarean delivery improves neonatal outcomes in FGR with abnormal Doppler findings remains limited. Additionally, absent end-diastolic flow (AEDF) often coexists with other complications such as maternal hypertension, congenital malformations, and other obstetrical factors. At the same time, the availability and level of neonatal intensive care may also influence observed neonatal outcomes, particularly in resource-constrained settings [2].

This study aims to elucidate the relationship between mode of delivery and perinatal outcomes in pregnancies complicated by AEDF. The study addresses a relatively specific and underexplored clinical question: whether mode of delivery is independently associated with an adverse perinatal outcome after accounting for gestational age, birth weight, and maternal factors at delivery.

## Materials and methods

This exploratory prospective observational cohort study was conducted between July 2019 and December 2020 at the Department of Obstetrics and Gynecology of a tertiary care center in northern India. Pregnant women with singleton gestations beyond 28 weeks with evidence of AEDF in the umbilical artery on fetal Doppler were eligible for inclusion. Consecutive patients meeting the inclusion criteria were recruited after obtaining informed consent. Women with multiple gestations, known fetal congenital anomalies, chromosomal abnormalities, or those unwilling to undergo recommended obstetric interventions were excluded. Ethical approval was obtained from the Institutional Ethics Committee, and the study was conducted in accordance with the Declaration of Helsinki.

All participants underwent standardized clinical evaluation, including a detailed obstetric history, physical examination, and relevant laboratory investigations. Fetal surveillance was performed using a biophysical profile and non-stress test, as well as Doppler studies of the umbilical artery and the ductus venosus.

Management decisions regarding the timing and mode of delivery were individualized based on gestational age, fetal surveillance findings, and maternal condition, in line with institutional protocols and the treating obstetrician. The time interval between AEDF diagnosis and delivery and the indications for cesarean delivery, including abnormal fetal surveillance, previous cesarean section, malpresentation, and maternal indications, were documented. A trial of labor was considered in selected cases with reassuring fetal parameters.

The primary outcome was a composite perinatal outcome defined as the presence of one or more of the following: cord pH < 7.1, five-minute Apgar score < 7, feeding intolerance, neonatal sepsis, necrotizing enterocolitis, or neonatal mortality. Secondary variables included gestational age at delivery, birth weight, maternal hypertension, FGR, oligohydramnios, and abnormal fetal surveillance findings.

Data were analyzed using IBM SPSS Statistics for Windows, Version 22 (Released 2013; IBM Corp., Armonk, New York). Continuous variables were expressed as means with standard deviations or medians with interquartile ranges, as appropriate, and categorical variables as frequencies and percentages. Group comparisons were performed using the chi-square test or Fisher's exact test for categorical variables and the Student's t-test or Mann-Whitney U test for continuous variables, as appropriate.

To adjust for confounding, multivariate logistic regression analysis was performed. Variables included in the model were gestational age at delivery, birth weight, mode of delivery, FGR, abnormal non-stress test, and maternal hypertension. Adjusted odds ratios with 95% confidence intervals were calculated.

## Results

A total of 71 women with singleton pregnancies complicated by AEDF were included in the study (Table 1). The mean maternal age was approximately 29.8 ± 5.2 years. The mean gestational age at diagnosis of AEDF was approximately 32.9 ± 2.8 weeks (27.4-38), and the mean gestational age at delivery was 33.43 ± 6.8 weeks. Hypertensive disorders were present in most cases, and FGR was observed in nearly three-quarters of the cohort. The average duration between diagnosis of AEDF and delivery was approximately eight days.

**Table 1:** The demographic profile of women with AEDF in the umbilical artery Doppler is shown. Values are expressed as mean ± standard deviation or number (percentage). AEDF: absent end-diastolic flow; FGR: fetal growth restriction; BOH: bad obstetric history.

Characteristics	Mean ± SD (n = 71)
Age (years)	29.8 ± 5.2 (19-45)
Primiparous	27 (38%)
Gestation at enrollment	32.67 ± 3.08 (28.1-38.4)
Gestation at AEDF detection (weeks)	32.9 ± 2.8 (27.4-38)
Previous obstetric history (n)%	
FGR	13 (18.3%)
AEDF	03 (4.2%)
BOH	16 (22.5%)
Medical and surgical disorders (n)%	34 (47.8%)
Chronic hypertension	18 (52.9%)
Renal	02 (2.8%)
Others	14 (19.7%)
Hypertensive disorders of pregnancy (n)%	66 (93%)
Chronic hypertension	18 (25.3%)
Preeclampsia	48 (61.9%)
FGR	53 (74.6%)
FGR and preeclampsia	16 (22.5%)
Antihypertensive usage (n)%	50 (70.4%)
One drug	33 (46.5%)
Two drugs	17 (23.9%)

Cesarean section was the predominant mode of delivery, performed in 58 out of 71 women, accounting for 81.7%. Among these, both elective and emergency cesarean sections were performed, with abnormal fetal surveillance being the most common indication for emergency procedures. A trial of labor was attempted in 26 women, of whom 13 achieved vaginal delivery, while the remaining women required emergency cesarean section (Table 2).

**Table 2:** The table summarizes the fetal monitoring, delivery details, and perinatal outcomes. AEDF: absent end-diastolic flow; NST: non-stress test; DV: ductus venosus; FGR: fetal growth restriction; CS: cesarean section; NICU: neonatal intensive care unit; APO: adverse composite perinatal outcome.

Fetal surveillance and mode of delivery	Mean ± SD; (n)%; (n = 71)
Gestation at AEDF detection (weeks)	32.91 ± 2.8 (27.4-38)
Duration of AEDF (days)	
1-7 days	61
8-14 days	4
5-21 days	4
22-28 days	1
36-42 days	1
Abnormal NST at termination	45 (63.4%)
Abnormal DV flow during hospital stay	5 (7%)
Gestation at termination (weeks)	33.43 ± 6.8
Steroid coverage	
Incomplete (less than four doses)	19 (26.8%)
Covered (four doses of dexamethasone)	52 (73.2%)
Attempt of vaginal delivery	26 (32.4%)
Final mode of delivery	
Vaginal delivery	13 (18.3%)
Cesarean sections	58 (81.7%)
Emergency (categories 1 and 2)	
Without the attempt of labor	46 (79.3%)
With the attempt of labor	13 (18.3%)
Elective/categories 3 and 4	12 (16.9%)
Indication for cesarean section	
Pathological cardiotocograph	38 (53.5%)
Severe oligohydramnios with FGR	2 (2.8%)
Previous CS	4 (5.6%)
Abruption	2 (2.8%)
Breech	9 (12.6%)
Non-progress of labor/meconium staining with poor bishops/placenta previa	3 (1 each) (4.2%)
Perinatal outcomes	
Birth weight (grams)	
<1,000	9 (12.6%)
1,000-1,500	35 (49.2%)
1.501-2,000	19 (26.7%)
2,001-2,500	6 (8.4%)
>2,501	2 (2.8%)
Gender	
Boy	34 (47.95%)
Girl	37 (52.1%)
APO	37 (52.1%)
Cord pH < 7.1	4 (5.6%)
Apgar at five minutes < 7	5 (7%)
Necrotizing enterocolitis	2 (2.8%)
Feed intolerance	31 (43.7%)
Sepsis	2 (29.6%)
Mortality	9 (12.7%)

Adverse composite perinatal outcomes (APOs) were observed in 37 neonates, representing 52% of the cohort. Feed intolerance and neonatal sepsis were among the most frequently observed issues. There were nine neonatal deaths, all occurring in infants with birth weights of less than 1.5 kg.

When outcomes were analyzed according to mode of delivery, a higher proportion of adverse outcomes was noted among women undergoing emergency cesarean section without a trial of labor. However, among women who underwent a trial of labor, the incidence of adverse outcomes was similar between those who delivered vaginally and those requiring cesarean section during labor.

Univariable analysis was performed. Lower gestational age at delivery and birth weight less than 1.5 kg were significantly associated with higher rates of adverse composite outcomes. Additionally, abnormal fetal surveillance findings at the time of termination/delivery were significantly associated with poor perinatal outcomes (Table 3).

**Table 3:** Univariable analysis of factors associated with adverse perinatal outcome. A p-value of <0.05 was considered significant by the chi-square test/Fisher exact test and is highlighted by *. AEDF: absent end-diastolic flow, CS: cesarean section.

Variable	Adverse perinatal outcome	No adverse perinatal outcome	OR (95% CI)	Chi-square statistic	p value
Birth weight < 1.5 kg (vs. >1.5 kg)	33 (75%)	11 (25%)	24 (6.0-95.45)	27.325	<0.001*
Gestational age at delivery <34 weeks (vs. ≥34 weeks)	27 (77.1%)	8 (22.9%)	10.13 (3.40-30.16)	19.304	<0.001*
Abnormal non-stress test					
Categories 2 and 3 (vs. category 1)	28 (30.8%)	17 (69.2%)	3.706 (1.326-10.360)	6.522	0.014*
Duration of AEDF >7 days (vs. ≤7 days)	8 (80%)	2 (20%)	4.71 (0.92-24.04)	3.997	0.85
Hypertension present (vs. absent)	29 (55.8%)	23 (44.2%)	2.16 (0.733-6.37)	1.994	0.158
Mode of delivery CS (vs. vaginal delivery)	32 (55.2)	26 (44.8)	2.77 (0.77-10.03)	2.53	0.112

To overcome the effect of confounding, we also did a multivariate logistic regression analysis of adverse perinatal outcomes. While abnormal cardiotocography (CTG) appeared to be an intermediate indicator of adverse perinatal outcome, the associated variables considered in the logistic regression included the following: birth weight, gestational age (<34 weeks), duration of AEDF (≥7 days), and mode of delivery (Table 4).

**Table 4:** Multivariable regression analysis for predictors of adverse perinatal outcome. Model statistics used: Model chi-square = 33.081, df = 4, p < 0.001; Nagelkerke R² = 0.497. Reference category: No adverse perinatal outcome. BW: birth weight; AEDF: absent end-diastolic flow; CS: cesarean section.

Variable	Adjusted OR	95% CI	p value
Birth weight (BW)	11.839	2.412-58.109	0.002
Gestational age at termination	2.619	0.657-10.438	0.172
Duration of AEDF (>=7 days)	2.086	0.330-13.184	0.434
Mode of delivery Cs	1.331	0.273-6.492	0.724

Birth weight was the only independent predictor of adverse perinatal outcome (OR 11.84; 95% CI: 2.41-58.11; p = 0.002) in our analysis. Gestational age at termination (p = 0.172), duration of AEDF ≥7 days, and mode of delivery (p = 0.724) did not show an independent association with adverse outcome. Increasing gestational age was associated with reduced risk, and higher birth weight had a protective effect. Mode of delivery was not independently associated with adverse outcomes after adjustment for confounding variables.

## Discussion

Overall, a high cesarean delivery rate was observed in our cohort, reflecting the high-risk nature of pregnancies complicated by AEDF and the frequent presence of fetal or obstetrical indications for operative delivery. This is consistent with FIGO guidance, which notes that cesarean delivery rates exceeding 80% have been reported when delivery is indicated in severe early-onset FGR [2]. In our cohort, 77% of women underwent direct cesarean delivery without a trial of labor, primarily because of acute fetal distress and malpresentation, while only 26 women underwent a trial of labor. Of these, approximately half delivered vaginally (18.3% of the overall cohort), whereas the remainder required cesarean delivery during labor.

Several observational studies have highlighted the importance of gestational age, birth weight, and concurrent fetal compromise in determining neonatal outcomes [6,7]. In a retrospective cohort of preterm growth-restricted fetuses, Baalbaki et al. found that intended cesarean delivery was not associated with a reduction in adverse neonatal outcomes, including among pregnancies with abnormal umbilical artery Doppler findings [8]. Similarly, Futterman et al. specifically evaluated pregnancies complicated by FGR and abnormal umbilical artery Doppler findings, highlighting the ongoing uncertainty regarding the optimal mode of delivery in this population [9]. The adverse outcomes among preterm infants are strongly influenced by the degree of prematurity and severity of growth restriction rather than by delivery route alone [4-8]. In a cohort of women with preeclampsia and absent or reversed end-diastolic flow, many adverse neonatal outcomes were explained predominantly by gestational age and FGR after adjustment, further emphasizing the contribution of underlying fetal maturity and growth status to neonatal morbidity [10].

In our cohort, perinatal outcomes were not independently associated with mode of delivery after adjustment for relevant clinical factors. Rather, birth weight emerged as the principal independent determinant of adverse perinatal outcome. These findings suggest that AEDF itself may not universally mandate cesarean delivery. In carefully selected pregnancies with reassuring fetal surveillance, absence of ductus venosus a-wave reversal, and appropriate clinical circumstances, a carefully monitored trial of labor may be considered. This interpretation is consistent with the limited observational literature suggesting that planned cesarean delivery does not necessarily confer a neonatal advantage in preterm FGR [8,9]. However, our findings should not be interpreted as evidence that vaginal delivery is generally safe in all pregnancies with AEDF. Rather, they suggest that the decision regarding route of delivery should incorporate the overall fetal condition and degree of maturity rather than AEDF in isolation.

Maternal hypertension influences placental function and fetal Doppler findings and commonly coexists with FGR. Approximately two-thirds of the participants in our study had hypertension. In univariate analysis, hypertension was associated with approximately 2.1-fold higher odds of adverse perinatal outcome, though it was not statistically significant. Maternal hypertension may have exerted its influence through gestational age at delivery, fetal growth, and birth weight. This interpretation is consistent with evidence that, in pregnancies complicated by preeclampsia and abnormal umbilical artery Doppler flow, gestational age and fetal weight account for a substantial proportion of adverse neonatal outcomes [10].

Prematurity and severity of fetal/placental compromise are therefore important potential mediators of adverse neonatal outcome, irrespective of whether birth occurs vaginally or by cesarean delivery [4-8,10]. In particular, the clinical consequences of prematurity may result in substantial neonatal morbidity even when delivery is undertaken because of appropriate fetal indications. This distinction is important when interpreting the association between emergency cesarean delivery and adverse outcomes in our cohort, because emergency delivery is more likely to occur in fetuses that are already clinically compromised. The duration of AEDF during the complete cardiac cycle and whether AEDF is intermittent or continuous may influence the adverse perinatal outcomes [11-14].

When we examined APOs, they were more frequent among emergency cesarean deliveries performed without a trial of labor. At face value, this could be interpreted as a disadvantage of surgical birth. However, this association is highly susceptible to confounding by indication: in most cases, the decision for emergency cesarean delivery was prompted by acute fetal compromise identified on non-stress testing or other clinical assessment. The indication for cesarean, therefore, represents a marker of pre-existing fetal compromise rather than necessarily an effect of cesarean delivery itself [2,8,9]. After adjusting for gestational age, birth weight, and duration of AEDF in a multivariable regression analysis, mode of delivery was not an independent predictor of adverse outcome. Instead, birth weight emerged as the principal determinant. This finding reinforces the possibility that the poorer outcomes observed after emergency cesarean delivery may primarily reflect the clinical condition that prompted delivery. Consequently, AEDF itself may not universally mandate cesarean delivery. A poor Bishop score may necessitate induction of labor, which in turn increases the odds of cesarean delivery [15]. However, Baalbaki et al. inferred that planned cesarean delivery does not necessarily eliminate the neonatal risk of a compromised fetus [8]. Therefore, a carefully monitored trial of labor in appropriately selected women with reassuring fetal surveillance may be considered. However, it is important to recognize that the evidence remains observational and that a low threshold for cesarean delivery is appropriate if fetal surveillance testing becomes non-reassuring.

Nevertheless, we do not suggest that cesarean delivery is unwarranted in all cases of AEDF. Rather, our findings highlight the importance of individualized decision-making that incorporates fetal weight, gestational age, and ongoing fetal surveillance. In pregnancies where fetal surveillance remains reassuring, ductus venosus flow is preserved, and gestational age is more advanced, a carefully monitored trial of labor may be considered in appropriately selected cases. In addition, avoiding unnecessary cesarean delivery may reduce maternal morbidity and the potential long-term reproductive consequences associated with repeated cesarean births. In resource-constrained settings, individualized planning should also consider the availability of neonatal intensive care, although resource limitations should not be used to justify compromising indicated fetal surveillance or timely delivery. A comprehensive discussion with parents is necessary to decide on the mode of delivery, including the possibility of intrapartum cesarean delivery if fetal status deteriorates. Continuous intrapartum fetal monitoring and readiness for expedited operative delivery are essential when a trial of labor is undertaken.

However, our study is not without limitations. The observational design precludes causal inference, and despite multivariable adjustment, residual confounding may persist. The relatively small sample size and single-center design may limit generalizability. Moreover, access to neonatal intensive care resources varied, potentially influencing outcomes. This is a practical limitation, especially for LMIC settings. The lack of randomization and small sample size make it difficult to compare outcomes between vaginal and cesarean delivery at different gestational ages. Furthermore, the relatively small number of women undergoing a trial of labor limits the precision with which the safety of vaginal delivery can be assessed. However, regression analysis helped to clarify the independent association of birth weight with adverse outcome in our population. For fetal surveillance, we used non-stress testing, biophysical profiles, and Doppler imaging, as computerized cardiotocography (cCTG) was not available during the study period.

Nevertheless, the findings contribute to a nuanced understanding of delivery planning in high-risk pregnancies complicated by umbilical artery AEDF. Birth weight rather than the mode of delivery was associated with perinatal outcome. However, these findings should be interpreted cautiously as hypothesis-generating rather than as evidence to routinely recommend vaginal delivery in AEDF. They provide a basis for further prospective, adequately powered studies evaluating whether carefully selected pregnancies with reassuring fetal surveillance can safely undergo a trial of labor.

## Conclusions

In pregnancies complicated by AEDF, perinatal outcomes are influenced by birth weight, gestational age, and fetal condition at the time of delivery. Although limited by observational design, our study suggests that mode of delivery may not significantly influence outcomes after accounting for the above factors. A carefully selected trial of labor may be considered in appropriate cases with reassuring fetal surveillance and preserved ductus venosus a-wave flow, provided that continuous intrapartum monitoring and facilities for expedited cesarean delivery are available. Comprehensive counseling, considering both maternal and fetal factors and available healthcare resources, should be undertaken, and individualized decision-making with the couple should be documented. The findings should not be interpreted as evidence against cesarean delivery when fetal surveillance is non-reassuring or other obstetric indications are present.
